# D-Allulose Production from D-Fructose by Permeabilized Recombinant Cells of *Corynebacterium glutamicum* Cells Expressing D-Allulose 3-Epimerase *Flavonifractor plautii*

**DOI:** 10.1371/journal.pone.0160044

**Published:** 2016-07-28

**Authors:** Chul-Soon Park, Taeyong Kim, Seung-Hye Hong, Kyung-Chul Shin, Kyoung-Rok Kim, Deok-Kun Oh

**Affiliations:** 1 Department of Bioscience and Biotechnology, Konkuk University, Seoul, 05029, South Korea; 2 Department of Food Science and Technology, Sejong University, Seoul, 05006, South Korea; Virginia Tech, UNITED STATES

## Abstract

A d-allulose 3-epimerase from *Flavonifractor plautii* was cloned and expressed in *Escherichia coli* and *Corynebacterium glutamicum*. The maximum activity of the enzyme purified from recombinant *E*. *coli* cells was observed at pH 7.0, 65°C, and 1 mM Co^2+^ with a half-life of 40 min at 65°C, *K*_m_ of 162 mM, and *k*_cat_ of 25280 1/s. For increased d-allulose production, recombinant *C*. *glutamicum* cells were permeabilized via combined treatments with 20 mg/L penicillin and 10% (v/v) toluene. Under optimized conditions, 10 g/L permeabilized cells produced 235 g/L d-allulose from 750 g/L d-fructose after 40 min, with a conversion rate of 31% (w/w) and volumetric productivity of 353 g/L/h, which were 1.4- and 2.1-fold higher than those obtained for nonpermeabilized cells, respectively.

## Introduction

The International Society of Rare Sugars (ISRS) stated at the 2014 Rare Sugar Symposium that ‘d-psicose’ should be referred to as ‘d-allulose’ and use of the ‘d-psicose’ term should be discontinued because d-allulose is an isomerized product of d-allose. d-Allulose (d-psicose, d-ribo-2hexulose) is a rare sugar that is present in small amounts as a non-fermentable component of commercial carbohydrates [1] and as a free sugar in agricultural products [2]. This sugar has attracted a great deal of attention in the field of functional foods owing to its health benefits. d-Allulose is used as a low-calorie sweetener and as a functional sugar for diabetes because it does not contribute calories and exhibits hypoglycemic, hypolipidemic, and antioxidant activities [3–6].

d-Allulose has been produced from d-fructose by the reactions of biocatalysts, including d-tagatose 3-epimerases (DTEases) from *Pseudomonas cichorii* [7] and *Rhodobacter sphaeroides* [8]; d-allulose 3-epimerases (DAEases) from *Agrobacterium tumefaciens* [9], *Clostridium* sp. [10], *Clostridium bolteae* [11], *Clostridium cellulolyticum* [12], *Clostridium scindens* [13], *Desmospora* sp. [14], *Dorea* sp. [15], *Ruminococcus* sp. [16], and *Treponema primitia* [17]; whole cells of *R*. *sphaeroides* [18] and *Sinorhizobium* sp. [19]; and whole recombinant cells of *Escherichia coli* expressing DAEases from *C*. *bolteae* [11], *C*. *cellulolyticum* [12], and *A*. *tumefaciens* [20]. Whole cells show greater stability and resistance to environmental perturbations than enzymes. Moreover, cells eliminate the need for purification steps, such as cell lysis, precipitation, and dialysis, and therefore the reactions are more commercially feasible [20]. Recombinant *E*. *coli* cells are suitable for d-allulose production because the specific productivity of these cells is significantly higher than that of wild-type cells. However, d-allulose produced by *E*. *coli* is limited in its use as a food additive because *E*. *coli* is not a generally recognized as safe (GRAS) host [21]. This problem can be solved by transferring the DAEase gene to a GRAS host such as *Corynebacterium glutamicum*. The organism is typically used for the industrial production of fine chemicals, owing to its ease of gene manipulation, rapid growth, and ability to grow to high density on cheap growth media [22]. In addition, its cell wall is structurally stable due to a lipid-rich outer layer mostly consisting of mycolic acid [23, 24]. However, the production of d-allulose using a GRAS host has not yet been attempted.

Permeabilized cells treated with detergents such as cetyl trimethylammonium bromide (CTAB), Tween 20, Tween 80, and Triton X-100 [18, 25]; solvents such as acetone, chloroform, ethanol, methanol, and toluene [19, 26, 27]; salts such as NaCl and MgCl_2_ [28]; and chemicals such as dimercaptosuccinic acid, EDTA, and polyethylenimine [28] have been used in whole-cell bioprocesses because they increase the production of metabolites by enhancing the transfer of reaction substrates and products across the cell membrane [26, 29]. Although penicillin has been used in glutamic acid fermentation to permeabilize *C*. *glutamicum* [30, 31], cell permeabilization with antibiotics has not yet been applied to whole-cell bioprocesses.

In the present study, a putative DAEase gene from *F*. *plautii* was cloned and expressed in *E*. *coli* and *C*. *glutamicum*. The biochemical properties, including metal ions, pH, temperature, and kinetic parameters, of the enzyme purified from recombinant *E*. *coli* cells were investigated. To increase the production of d-allulose from d-fructose, recombinant *C*. *glutamicum* cells expressing DAEase from *F*. *plautii* were permeabilized using several types of substances, including antibiotics, detergents, and solvents; and the most effective antibiotic, detergent, and solvent for d-allulose production were selected. The most effective combined permeabilizers were determined by treatment with the selected permeabilizers in combination. The reaction conditions, including pH, temperature, metal ions, and the concentrations of cells and substrate, were optimized for the permeabilized cells. Under the optimized conditions, the increased production of d-allulose from d-fructose was achieved.

## Materials and Methods

### Materials

d-Allulose, d-fructose, penicillin, ethambutol, ethionamide, and isoniazid standards were purchased from Sigma (St. Louis, MO, USA). Bio-LC grade sodium hydroxide solution was purchased from Fisher Scientific (Hanover Park, IL, USA). All of the restriction enzymes were purchased from New England Biolabs (Hertfordshire, UK, USA). Solvents and detergents were purchased from Santa Cruz Biotechnology (Santa Cruz, CA, USA).

### Cloning and gene expression

The genomic DNA from *F*. *plautii* ATCC 29863 (ATCC, Manassas, USA), *E*. *coli* ER2566 (New Englands Biolab, Hertfordshire, UK), and pET15b plasmid (Novagen, Madison, WI) were used as the sources of the DAEase gene, host cells, and expression vector. The gene encoding the putative DAEase was amplified by PCR using *F*. *plautii* genomic DNA as a template. The primer sequences used for gene cloning were based on the DNA sequence of the putative DAEase from *F*. *plautii* (GenBank accession number EHM40452.1). Forward (5′- CATATGAACCCGATTGGAATGCACTAC-3′) and reverse primers (5′- CTCGAGTTACGCGGTCAGCTCCTTGAGG-3′) were designed to introduce the underlined *Nde*I and *Xho*I restriction sites, respectively, and were synthesized by Bioneer (Daejon, Korea). The DNA fragment amplified by PCR was purified using a PCR purification kit (Promega, Madison, WI, USA) and inserted into the pET15b vector digested with the same restriction enzymes. The resulting plasmid was transformed into *E*. *coli* strain ER2566 using an electroporator (MicroPulser, Bio-Rad, Hercules, CA, USA). The transformed *E*. *coli* was plated on Luria-Bertani (LB) agar containing 25 μg/mL ampicillin. An ampicillin-resistant colony was selected, and the plasmid DNA from the transformant was isolated with a plasmid purification kit (Promega). DNA sequencing was carried out at the Macrogen facility (Seoul, Korea). Gene expression was estimated by both SDS-PAGE and enzyme activity assay.

*C*. *glutamicum* ATCC 13032 (ATCC, Manassas, USA), and *E*. *coli*−*C*. *glutamicum* shuttle expression vector pEKEx2 (Juelich Research Centre, Juelich, Germany) were used as the sources of host cells and expression vector, respectively. The DAEase gene from *F*. *plautii* was ligated into the expression vector pEKEx2. A ribosomal binding site (rbs) was encoded upstream of the DAEase gene, which was amplified by PCR using the template vector pET15b from *E*. *coli*. The primer sequences used for gene cloning were based on the DNA sequence of *F*. *plautii* DAEase. Forward (5′-CTGCAGAAAGGAGATATAGATGAACCCGATTGGAATGCACTACGGC-3′) and reverse primers (5′-GTCGACTTACGCGGTCAGCTCCTTGAG-3′) were designed to introduce the underlined *Pst*I and *Sal*I restriction sites, respectively, and were synthesized by Bioneer. The amplified DNA fragment obtained by PCR was purified and inserted into the pEKEx2 vector digested with the same restriction enzymes. The resulting plasmid was transformed into *C*. *glutamicum* strain ATCC 13032 using an electroporator. The transformed *C*. *glutamicum* cells were plated on brain-heart infusion (BHI) agar containing 15 *μ*g/mL kanamycin. A kanamycin-resistant colony was selected, and the plasmid DNA from the transformant was isolated with a plasmid purification kit (Promega). DNA sequencing was carried out at the Macrogen facility.

### Culture conditions

Recombinant *E*. *coli* containing the DAEase/pET15b gene from *F*. *plautii* was cultivated in a 2 L flask containing 500 mL of LB medium and 25 μg/mL ampicillin at 37°C with shaking at 200 rpm. When the optical density of the bacterial culture at 600 nm reached 0.6, 0.1 mM isopropyl-β-d-thiogalactopyranoside (IPTG) as a final concentration was added to the culture to induce DAEase expression, and the culture was then incubated with shaking at 150 rpm at 16°C for 16 h to express the enzyme.

Recombinant *C*. *glutamicum* containing the DAEase/pEKEx2 gene from *F*. *plautii* was cultivated in a 2 L flask containing 500 mL of BHI medium and 15 μg/mL kanamycin at 30°C with shaking at 200 rpm. When the optical density of bacteria culture at 600 nm reached 0.6, 1.0 mM IPTG and 20 mg/L penicillin as final concentrations were added to the culture to induce DAEase expression and to injure the peptidoglycan layers of *C*. *glutamicum* cell wall, respectively. For enzyme expression, the culture was further incubated with shaking at 200 rpm at 30°C for 20 h.

### Enzyme preparation

Recombinant *E*. *coli* expressing *F*. *plautii* DAEase was harvested from the culture broth by centrifugation at 8,000 × *g* for 30 min at 4°C, and then washed twice with 0.85% NaCl. Washed recombinant cells were resuspended in 50 mM phosphate buffer (pH 7.0) containing 300 mM NaCl and 1 mg/mL lysozyme. Resuspended cells were disrupted using a sonicator on ice for 2 min. Unbroken cells and cell debris were removed by centrifugation at 13000*g* for 20 min at 4°C, and the supernatant was filtered through a 0.45 μm pore size filter. All purification steps were carried out at 4°C with a Profinia protein purification system (Bio-Rad). The filtrate was loaded onto an immobilized metal ion affinity chromatography (IMAC) cartridge (Bio-Rad, Hercules, CA, USA), which was previously equilibrated with 50 mM phosphate buffer (pH 8.0). The bound protein was eluted with a linear gradient between 10 to 500 mM imidazole at a flow rate of 1 mL/min. The eluent was collected and loaded immediately onto a Bio-Gel P-6 desalting cartridge (Bio-Rad), previously equilibrated with 50 mM piperazine-*N*,*N*′-bis(2-ethanesulfonic acid) buffer (PIPES) (pH 7.0). The loaded protein was eluted with 50 mM PIPES buffer (pH 7.0) at a flow rate of 1 mL/min. The active fractions were collected and dialyzed against 50 mM PIPES buffer (pH 7.0) for 16 h. The solution resulting from dialysis was used as the purified enzyme.

### Determination of molecular mass

The subunit molecular mass of *F*. *plautii* DAEase was determined by SDS-PAGE under denaturing conditions, using a ladder of pre-stained proteins (MBI fermentas, Hanover, MD, USA) as references. All protein bands were stained with Coomassie Blue for visualization. The molecular mass of the native enzyme was investigated by gel-filtration chromatography using a Sephacryl S-300 preparative-grade column HR 16/60 (GE Healthcare, Piscataway, NJ, USA). The purified enzyme was loaded onto the column and eluted with 50 mM Tris-HCl (pH 7.5) buffer containing 150 mM NaCl at a flow rate of 1mL/min. The column was calibrated with ferritin (440 kDa), catalase (206 kDa), aldolase (158 kDa), and conalbumin (75 kDa) as reference proteins (GE Healthcare). The molecular mass of the native enzyme was calculated by comparing with the migration length with that of the reference proteins.

### Determination of specific activity and kinetic parameters

One unit (U) of enzyme activity for d-fructose was defined as the amount of enzyme required to liberate 1 μmol of d-allulose per min at pH 7.0 and 65°C. The specific activity (U/mg) was defined as the amount of monosaccharide produced per enzyme amount per unit reaction time. Various concentrations of d-fructose and d-allulose (5−80 mM) were used to decide the kinetic parameters for the enzyme. *K*_m_ (mM) and *k*_cat_ (1/s) were determined by the Lineweaver-Burk plot derived from the Michaelis-Menten equation. The catalytic constant, *k*_cat_, was calculated by dividing the subunit molecular mass by the DAEase concentration.

### Preparation of permeabilized cells

To prepare permeabilized *C*. *glutamicum* cells, cells were harvested from the culture broth by centrifugation at 13,000 × *g* for 20 min at 4°C and then washed twice with 0.85% sodium chloride solution. The washed cells were resuspended in solutions containing 2 and 5 mg/L penicillin, and 2, 5, 50, and 100 mg/L ethambutol, ethionamide, and isoniazid as antibiotics; 0.2% and 0.5% (w/v) CTAB and Triton X-100, and 2% and 5% (w/v) Span 20, Span 80, Tween 20, Tween 40, and Tween 80 as detergents; and 10% and 20% (v/v) acetone, DMSO, ethanol, hexane, methanol, 1-propanol, iso-propanol, and toluene as solvents. The cell suspension was incubated at 4°C for 15 min and washed twice with 50 mM PIPES buffer (pH 7.0). The treated cells were used as permeabilized cells for the production of d-allulose from d-fructose. Unless otherwise stated, the enzyme and cell reactions were performed at 65°C for 10 min in 50 mM PIPES buffer (pH 7.0) containing 50 mM d-fructose in the presence of 1 mM Co^2+^ with 0.5 U/mL enzyme and 7.5 g/L cells, respectively, as standard conditions.

### Effects of temperature, pH, and metal ions on DAEase activity

The effects of temperature and pH on the activity of DAEase, were investigated by varying the temperature from 30 to 70°C at a constant pH of 7.0 and by varying the pH from 6.0 to 8.5 at a constant temperature of 65°C under the standard conditions. To evaluate the effect of metal ions on enzyme activity, the enzyme assay was conducted after treatment with 1 mM ethylenediaminetetraacetic aicd (EDTA) at 4°C for 1 h or after the individual additions of 1 mM MnSO_4_, MnCl_2_, NiSO_4_, CoCl_2_, MgSO_4_, and FeCl_3_.

### Effects of temperature, pH, and metal ions on the activity of recombinant cells

The effects of temperature and pH on the activity of permeabilized cells were examined by varying the temperature from 50 to 75°C in 50 mM PIPES buffer at a constant pH of 7.5 and by varying the pH from 6.0 to 8.5 at a constant temperature of 65°C, respectively. The effect of metal ions on the production of d-allulose from d-fructose was evaluated by nonpermeabilized and permeabilized *C*. *glutamicum* cells expressing DAEase from *F*. *plautii*.

### Thermal inactivation for the enzyme in permeabilized *C*. *glutamicum* cells and the purified enzyme

The influence of temperature on the stability of the enzyme in permeabilized cells was investigated as a function of the incubation time by placing the cell solution at five different temperatures (45, 50, 55, 60, and 65°C) in 50 mM PIPES buffer (pH 7.5). The influence of temperature on enzyme stability was a function of incubation time by placing the enzyme solution at five different temperatures (45, 50, 55, 60, and 65°C) in 50 mM PIPES buffer (pH 7.0). Samples were withdrawn at specific time intervals and the activities were measured under standard conditions. The half-life of the enzyme was calculated using Sigma Plot 9.0 software (Systat Software, San Jose, CA).

### Optimization of cell and substrate concentrations, and d-allulose production under the optimized conditions

The optimal concentrations of cells and substrate for increased production of d-allulose from d-fructose by whole permeabilized cells were determined by varying the cell concentration from 1 to 15 g/L at a constant d-fructose concentration of 750 g/L, and by varying the substrate concentration from 50 to 750 g/L at a constant cell concentration of 10 g/L. The production of d-allulose from d-fructose by whole permeabilized cells or nonpermeabilized cells was performed in 50 mM PIPES buffer (pH 7.5) containing 10 g/L cells, 750 g/L d-fructose, and 1 mM Co^2+^ at 65°C for 1 h.

### Analytical methods

Cell mass was determined using a linear calibration curve of optical density at 600 nm verse dry cell weight. The OD_600_ values of culture broth were measured and then converted to the dry cell weight (1 OD_600_ = 0.42 g/L dry cell weight). The concentrations of monosaccharides, including d-fructose, d-allulose, d-sorbose, d-tagatose, d-xylulose, and d-ribulose, were determined using a Bio-LC system (Dionex ICS-3000, Sunnyvale, CA, USA) with an electrochemical detector and a CarboPac PA10 column. The column was eluted at 30°C with 200 mM sodium hydroxide at a flow rate of 1 mL/min.

## Results and Discussion

### Gene cloning, purification, and molecular mass determination of DAEase from *F*. *plautii*

**Fig 1:**
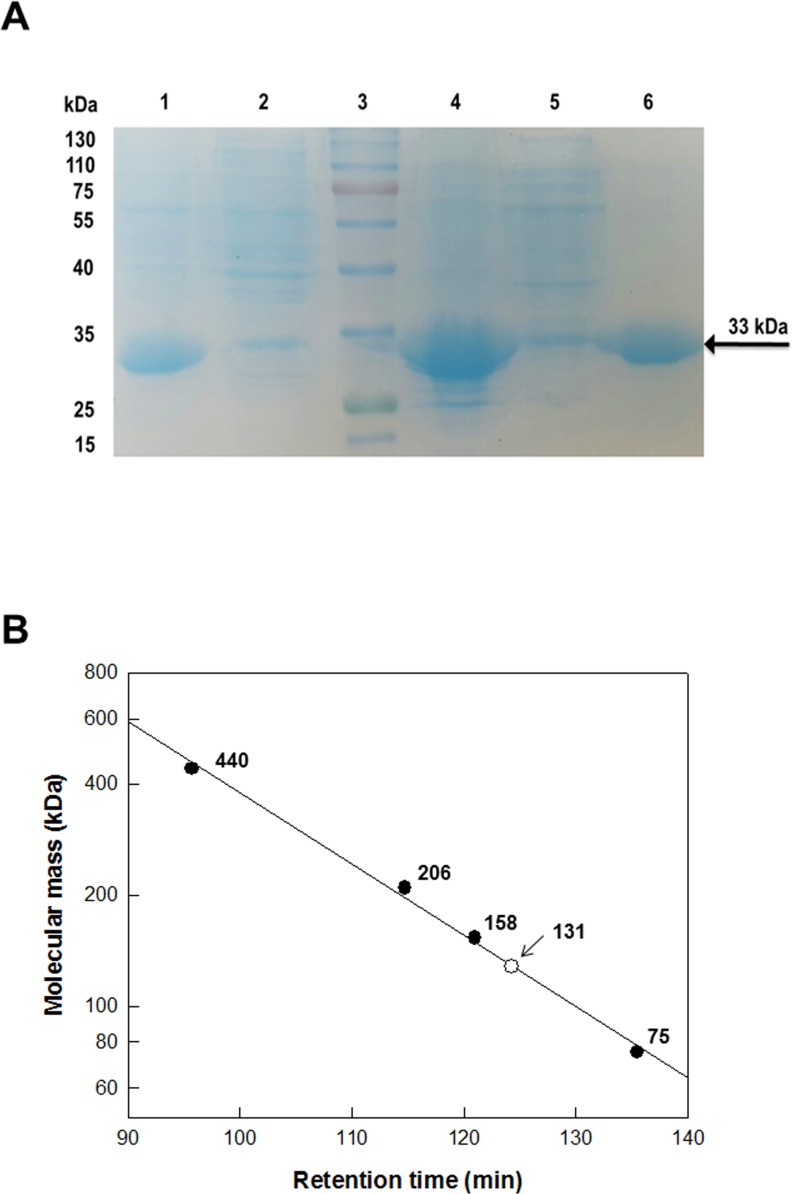
**(A) SDS-PAGE of DAEase from *F*. *plautii*.** Lanes: 1, pellet of *C*. *glutamicum* expressing DAEase; 2, crude extract of *C*. *glutamicum* expressing DAEase; 3, protein marker (130, 100, 75, 55, 40, 35, 25, and 17 kDa); 4, pellet of *E*. *coli* expressing DAEase; 5, crude extract of *E*. *coli* expressing DAEase; and 6, purified enzyme of *E*. *coli* expressing DAEase. **(B) Determination of total molecular mass for DAEase from *F*. *plautii* by gel filtration chromatography with reference proteins.** Ferritin (440 kDa), catalase (206 kDa), aldolase (158 kDa), and conalbumin (75 kDa); and DAEase from *F*. *plautii*.

The gene (885 bp) encoding *F*. *plautii* DAEase (GenBank Accession No. EHM40452.1) was cloned and expressed in *E*. *coli*. The expressed enzyme was purified as a soluble protein from a crude cell extract by His-trap affinity chromatography. The specific activity of the purified enzyme for d-allulose was 20 U/mg. The expressed protein analyzed by SDS-PAGE showed a single band with a molecular mass of approximately 33 kDa, consistent with the calculated value of 32,987 Da based on the 295 amino acids plus the hexa-histidine tag. The expression level of the protein in *C*. *glutamicum* was lower than that of *E*. *coli* (Lanes 1 and 4 of Fig 1A). However, the enzyme was expressed as a soluble protein with a similar level between two host cells in crude extracts (Lanes 2 and 5 of Fig 1A). The native enzyme existed as a tetramer with a molecular mass of 131 kDa as determined by gel filtration chromatography (Fig 1B and S1 Fig). The total molecular masses and association forms of DAEases and DTEases were 125–132 kDa and tetramers, respectively.

### Identification of the active-site residues of DAEase from *F*. *plautii*

The amino acid sequence of *F*. *plautii* DAEase was aligned with those of DTEase from *P*. *cichorii* [32] and DAEases from *A*. *tumefaciens* [33] and *C*. *cellulolyticum* [34], having the determined crystal structures (S2 Fig). The active site residues of *A*. *tumefaciens* DAEase were the same as those of *C*. *cellulolyticum* DAEase. The metal-binding residues were coordinated by Glu152, Asp185, His211, and Glu246 in *P*. *cichorii* DTEase; and Glu150, Asp183, His209, and Glu244 in *A*. *tumefaciens* DAEase. The metal-binding residues in *F*. *plautii* DAEase were absolutely conserved with Glu155, Asp188, His214, and Glu 250. The substrate-binding residues were Phe7, Trp15, Cys66, Leu108, Trp113, Glu158, His188, Arg217, and Phe248 in *P*. *cichorii* DTEase; and Tyr6, Trp14, Gly65, Ala107, Trp112, Glu156, His186, Arg215, and Phe248 in *A*. *tumefaciens* DAEase. Among the predicted substrate-binding residues of *F*. *plautii* DAEase, Trp15, Trp115, Glu161, His191, Arg220, and Phe252, were absolutely conserved across *P*. *cichorii* DTEase and *A*. *tumefaciens* DAEase. However, the other substrate-binding residues, including His7, Ser68, and Val110 in *F*. *plautii* DAEase, were different for the three enzymes.

### Biochemical properties of DAEase from *F*. *plautii*

The biochemical properties of *F*. *plautii* DAEase are compared with other reported DTEases and DAEases in Table 1. The maximum enzyme activity of the conversion of d-fructose to d-allulose by *F*. *plautii* DAEase was observed at 65°C (S3A Fig) and pH 7.0 (S3B Fig). The activities of DAEase from *Dorea* sp. [15] and DTEase from *R*. *sphaeroides* [8], were maximal at pH 6.0 and pH 9.0, respectively. The pH values of other DAEases and DTEases were maximal in the range of 7.0 to 8.0. The lowest and highest temperatures for maximal activity were 40°C for DTEase from *R*. *sphaeroides* [8] and 70°C for DAEases from *Dorea* sp. [15] and *T*. *primitia*. [17] The effect of metal ions such as MnSO_4_, MnCl_2_, NiSO_4_, CoCl_2_, MgSO_4_, and FeCl_3_ on the activity of *F*. *plautii* DAEase was evaluated (S3C Fig). The activity was highest in the presence of Co^2+^, and DAEase form *F*. *plautii* was not activated without divalent cations, indicating that the enzyme is metal-dependent. Most other DAEases [10–12, 14, 15, 17] also showed the highest activity in presence of Co^2+^. The half-life of DAEase from *C*. *cellulolyticum* at 60°C was 408 min [12], which was the highest reported thermal stability among the DAEases and DTEases that was 3.1-fold higher than that of DAEase from *F*. *plautii*. DAEase from *F*. *plautii* produced 239 g/L d-allulose from 750 g/L d-fructose at pH 7.0 and 65°C after 60 min, with a conversion rate of 32% and a productivity of 239 g/L/h (S4 Fig). This is the highest production of d-allulose ever reported. The conversion rates of d-fructose to d-allulose by DAEases were higher than those by DTEases.

**Table 1:** Biochemical properties of DAEases and DTEases.

Enzyme	pH	Temp. (°C)	Metal ions	Half-life at 60°C (min)	*k*_cat_ (1/s)		*K*_m_ (mM)		*k*_cat_/*K*_m_ (1/mM/min)		d-Allulose (g/L)[d-Fructose, g/L]	Reference
					d-Allulose	d-Fructose	d-Allulose	d-Fructose	d-Allulose	d-Fructose		
*A*. *tumefaciens* DAEase	8.0	50	Mn^2+^	4^*b*^	40	35	12	24	205	85	230 [700]	[9]
*Clostridium* sp. DAEase	8.0	65	Co^2+^	10^*c*^	537	273	228	279	141	59	120 [500]	[10]
*C*. *bolteae* DAEase	7.0	55	Co^2+^	45^*b*^	49	59	27	60	107	59	216 [750]	[11]
*C*. *cellulolyticum* DAEase	8.0	55	Co^2+^	408^*c*^	54	56	17	54	186	63	218 [750]	[12]
*C*. *scindens* DAEase	7.5	55	Mn^2+^	50^*b*^	31	9	28	40	65	9	NR	[13]
*Desmospora* sp. DAEase	7.5	60	Co^2+^	NR^*a*^	86	1,060	81	549	327	116	143 [500]	[14]
*Dorea* sp. DAEase	6.0	70	Co^2+^	60^*c*^, 30^*b*^	1,311	508	191	153	412	199	115 [500]	[15]
*P*. *cichorii* DTEase	7.5	60	None	NR	NR	NR	NR	NR	NR	NR	150 [780]	[7]
*R*. *sphaeroides* DTEase	9.0	40	Mn^2+^	NR	NR	NR	NR	NR	NR	NR	118 [700]	[8]
*Ruminococcus* sp. DAEase	7.5–8.0	60	Mn^2+^	96^*b*^	41	59	48	216	51	16	125 [500]	[16]
*T*. *primita* DAEase	8.0	70	Co^2+^	< 30^*b*^	503	292	209	279	144	63	138 [500]	[17]
*F*. *plautii* DAEase	7.0	65	Co^2+^	130^*b*^	421	342	162	323	156	64	239 [750]	This study

^*a*^NR, not reported.

^*b*^ and ^*c*^ represent that the half-life of each enzyme was measured without and with metal ions, respectively.

DAEases from *Desmospora* sp. [14] and *Dorea* sp. [15] exhibited the highest turnover number (*k*_cat_) for d-fructose and d-allulose, respectively. The catalytic efficiencies of DAEases for d-allulose were 2−3-fold higher than those for d-fructose except for *C*. *scindens* DAEase [13], which showed 7.2-fold higher catalytic efficiency for d-allulose than for d-fructose. Among the hexoketose substrates, the specific activity of *F*. *plautii* DAEase followed the order d-allulose > d-fructose > d-ribulose > d-xylulose > d-sorbose > d-tagatose (Table 2). The substrate specificity of *A*. *tumefaciens* DAEase followed the order d-allulose > d-fructose > d-tagatose > d-ribulose > d-xylulose > d-sorbose [9].

**Table 2:** Substrate specificity of *F*. *plautii* DAEase for hexoketose.

Ketose	Relative activity (%)
d-Allulose	100.0 ± 1.82
d-Fructose	41.1 ± 1.71
d-Tagatose	2.6 ± 0.07
d-Sorbose	6.9 ± 0.04
d-Xylulose	8.3 ± 0.16
d-Ribulose	10.5 ± 1.45

Data expressed as the mean of three separate experiments ± standard deviation.

### Permeabilization of recombinant *C*. *glutamicum* cells expressing DAEase from *F*. *plautii* for increased production of d-allulose

**Fig 2:**
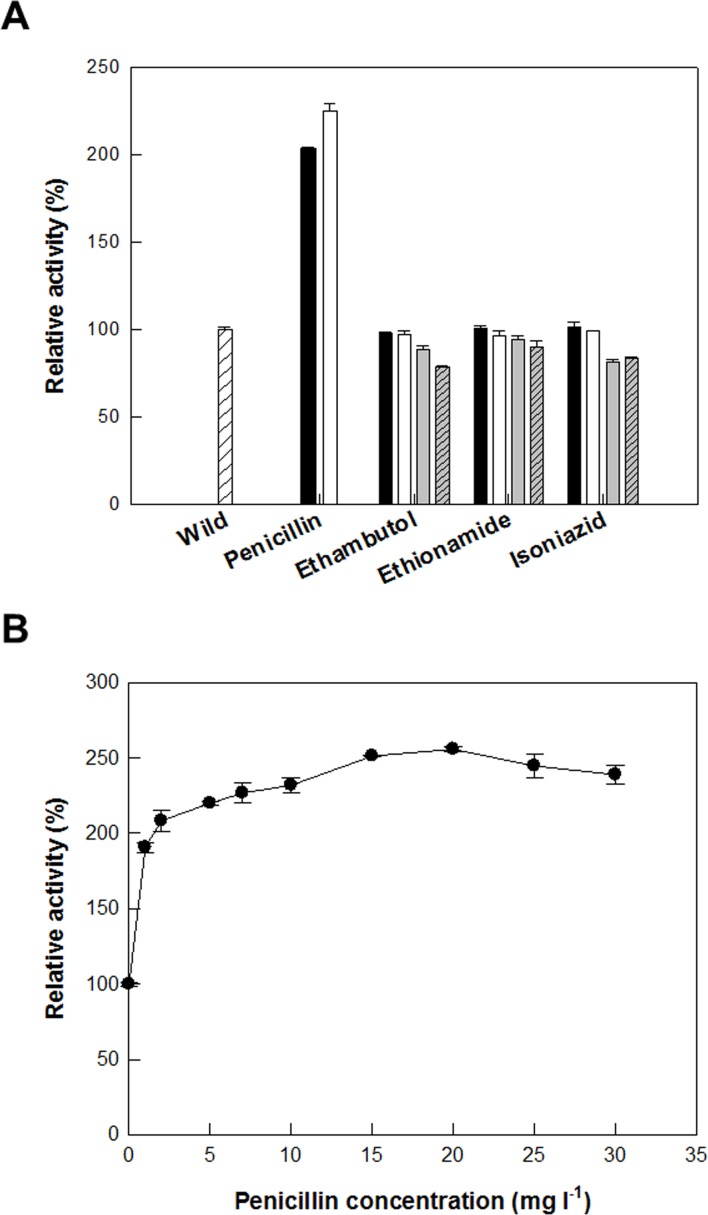
Effect of antibiotic treatment on the permeabilization of *C*. *glutamicum* expressing DAEase from *F*. *plautii* for the production of d-allulose from d-fructose. **(A) Effect of antibiotic treatment.** 0 (white bar with diagonal line), 2 (black bar), 5 (white bar), 50 (gray bar), and 100 mg/L (gray bar with diagonal line). **(B) Effect of penicillin concentration.** The reactions were performed in 50 mM PIPES buffer (pH 7.0) containing 7.5 g/L cells and 50 mM d-fructose at 65°C for 10 min. Data represent the means of three separate experiments and error bars represent the standard deviation.

**Fig 3:**
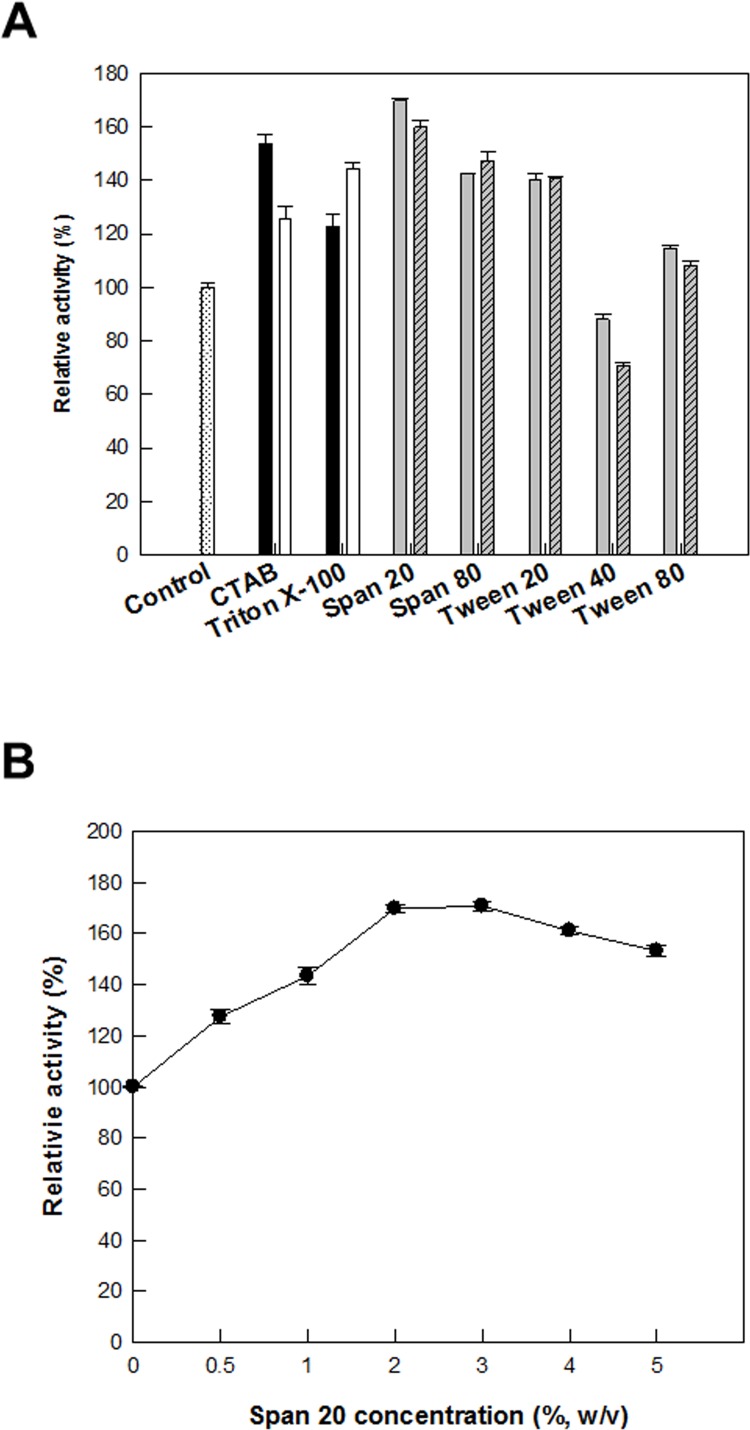
Effect of detergent treatment on the permeabilization of *C*. *glutamicum* expressing DAEase from *F*. *plautii* for the production of d-allulose from d-fructose. **(A) Effects of detergent treatment.** 0 (white bar with dotted line), 0.2 (black bar), 0.5 (white bar), 2 (gray bar), and 5% (w/v) (gray bar with diagonal line). **(B) Effect of span 20 concentration.** The reactions were performed in 50 mM PIPES buffer (pH 7.0) containing 7.5 g/L cells and 50 mM d-fructose at 65°C for 10 min. Data represent the means of three separate experiments and error bars represent the standard deviation.

**Fig 4:**
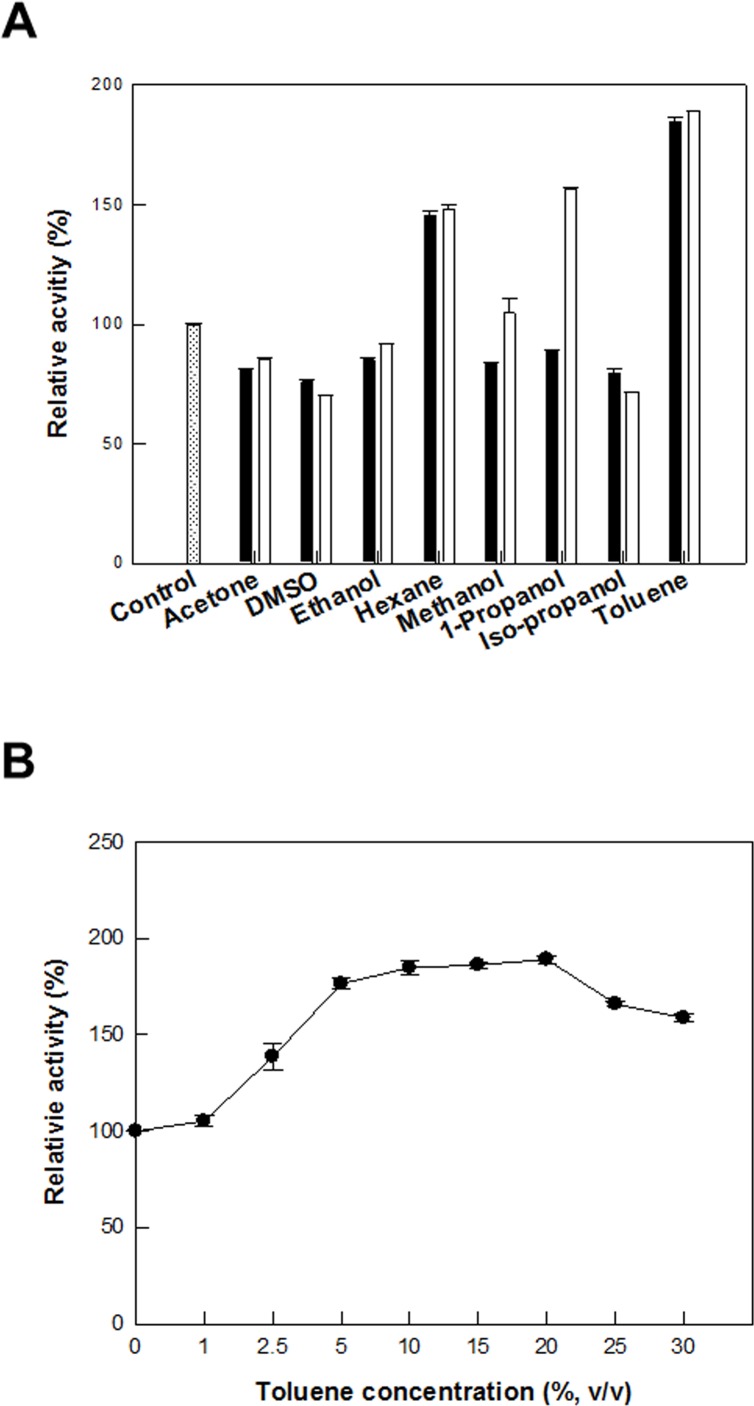
Effect of solvent treatment on the permeabilization of *C*. *glutamicum* expressing the DAEase from *F*. *plautii* for the production of d-allulose from d-fructose. **(A) Effect of solvent treatment.** 0 (white bar with dotted line), 10 (black bar), and 20% (v/v) (white bar). **(B) Effect of toluene concentration.** The reactions were performed in 50 mM PIPES buffer (pH 7.0) containing 7.5 g/L cells and 50 mM d-fructose at 65°C for 10 min. Data represent the means of three separate experiments and error bars represent the standard deviation.

To increase d-allulose production, recombinant *C*. *glutamicum* cells expressing DAEase from *F*. *plautii* were permeabilized by treatment with antibiotics, including ethambutol, ethionamide, isoniazid, and penicillin; detergents, including CTAB, Span 20, Span 80, Triton X-100, Tween 20, Tween 40, and Tween 80; and solvents, including acetone, DMSO, ethanol, hexane, methanol, 1-propanol, isopropanol, and toluene. Permeabilized cells treated with 5 mg/L penicillin, 2% (w/v) span 20, and 20% (v/v) toluene showed the highest activity among the antibiotics, detergents, or solvents, respectively, and showed 2.2-, 1.6-, and 1.7-fold higher activity than that obtained by non-treated cells, respectively (Figs 2A, 3A and 4A). The effects of the concentrations of penicillin, span 20, and toluene on the production of d-allulose from d-fructose were investigated. The production of d-allulose was maximal in permeabilized cells treated with 20 mg/L penicillin, 3% (w/v) span 20, and 20% (v/v) toluene (Figs 2B, 3B and 4B), and was 2.5-, 1.7-, and 1.8-fold higher than that by non-treated cells, respectively. Penicillin was used to permeabilize *C*. *glutamicum* during glutamic acid fermentation [31] and toluene was an efficient permeabilizer to *G*. *suboxydans* for the production of l-sorbose [35].

**Fig 5:**
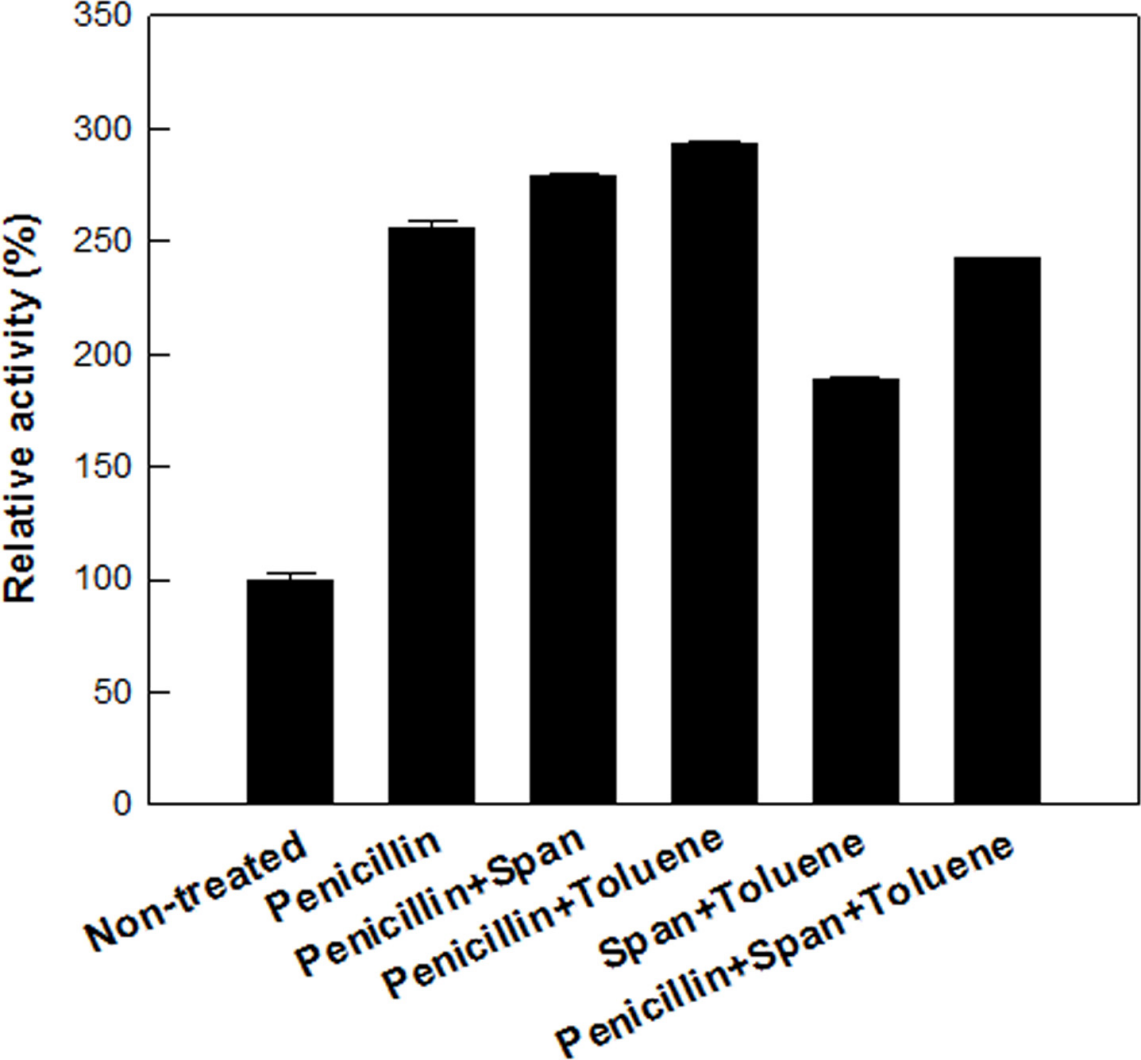
Effect of the combined treatment of permeabilizers on the permeabilization of *C*. *glutamicum* expressing DAEase from *F*. *plautii* for the production of d-allulose from d-fructose. The concentrations of penicillin, span 20, and toluene used were 20 mg/L, 2% (w/v), and 10% (v/v), respectively. The reactions were performed in 50 mM PIPES buffer (pH 7.0) containing 7.5 g/L cells and 50 mM d-fructose at 65°C for 10 min. Data represent the means of three separate experiments and error bars represent the standard deviation.

To determine the synergistic effect in d-allulose production, cells were treated with 20 mg/L penicillin, 3% (w/v) Span 20, and 20% (v/v) toluene in combination. Permeabilized cells treated with 20 mg/L penicillin and 20% (v/v) toluene showed the highest activity among cells permeabilized by the combined treatment, and exhibited 3-fold higher d-allulose production than that by non-treated cells (Fig 5). The increases in d-allulose production by permeabilized cells treated with efficient permeabilizers such as penicillin, Span 20, and toluene were not critical above 2 mg/L, 1% (w/v), and 5% (v/v), respectively, which were all lower concentrations than those used for the maximal production of d-allulose, respectively. Cells were treated with a combination of the permeabilizers using these concentrations (S5 Fig). Permeabilized cells treated with 2 mg/L penicillin and 5% (v/v) toluene also showed the highest activity among cells permeabilized with the combined treatment and lower d-allulose production than that by permeabilized cells treated with 20 mg/L penicillin and 20% (v/v) toluene. Thus, permeabilized cells treated with 20 mg/L penicillin and 20% (v/v) toluene were used for d-allulose production.

### Effects of temperature, pH, and metal ions on the production of d-allulose from d-fructose by permeabilized recombinant *C*. *glutamicum* cells

**Fig 6:**
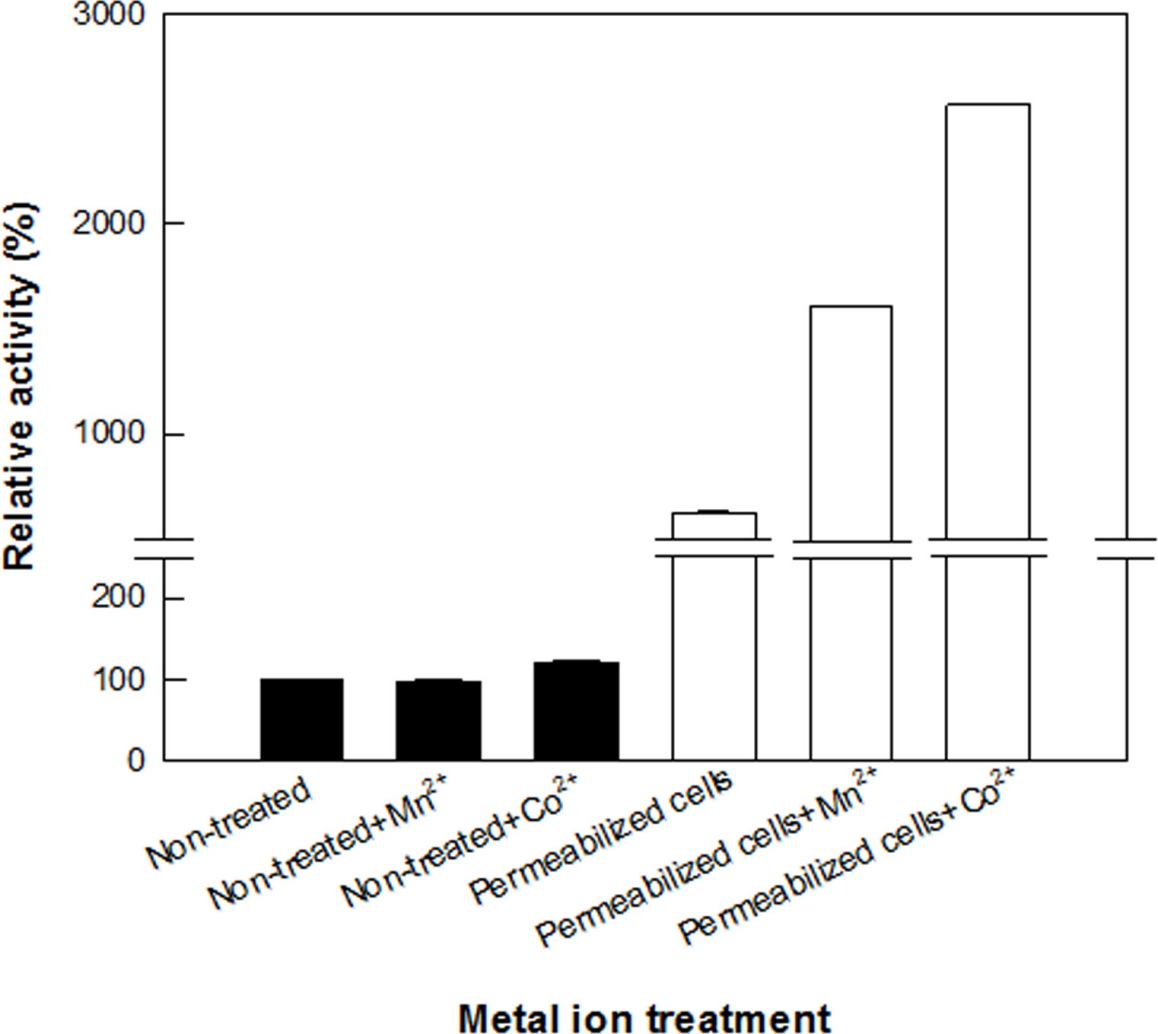
Effect of metal ions on the production of d-fructose to d-allulose by nonpermeabilized and permeabilized cells of *C*. *glutamicum* expressing DAEase from *F*. *plautii*. Production of d-allulose from d-fructose by nonpermeabilized cells (black bar) and permeabilized cells (white bar). The reactions were conducted in 50 mM PIPES buffer (pH 7.0) containing 7.5 g/L cells and 50 mM d-fructose in the presence of 1 mM metal ions at 65°C for 10 min. Data represent the means of three separate experiments and error bars represent the standard deviation.

The maximum activity of permeabilized *C*. *glutamicum* cells expressing DAEase from *F*. *plautii* for d-allulose production was observed at 65°C (S6A Fig) and pH 7.5 (S6B Fig). The reactions of whole recombinant *E*. *coli* cells expressing DAEase from *C*. *bolteae* [11], *C*. *cellulolyticum* [12], and *A*. *tumerfaciens* [20] were performed at pH 6.5 and 55°C, pH 8.0 and 55°C, and pH 8.5 and 60°C, respectively. Mn^2+^ and Co^2+^ strongly enhanced d-fructose epimerization by DAEases and DTEases. Thus, the effect of metal ions such as Mn^2+^ and Co^2+^ on the activity of d-allulose production was evaluated in nonpermeabilized and permeabilized *C*. *glutamicum* cells expressing DAEase from *F*. *plautii* (Fig 6). The activities of both recombinant nonpermeabilized and permeabilized *C*. *glutamicum* cells in the presence of Co^2+^ were higher than those in the presence of Mn^2+^. d-Allulose production by permeabilized cells in the presence of Co^2+^ was 25-fold higher than that by nonpermeabilized cells. The significantly increased production of d-allulose may be due to the improved contact of metal ions and the enzyme caused by cell permeabilization.

### Thermal inactivation for DAEase from *F*. *plautii* in permeabilized recombinant *C*. *glutamicum* cells and the purified DAEase

**Fig 7:**
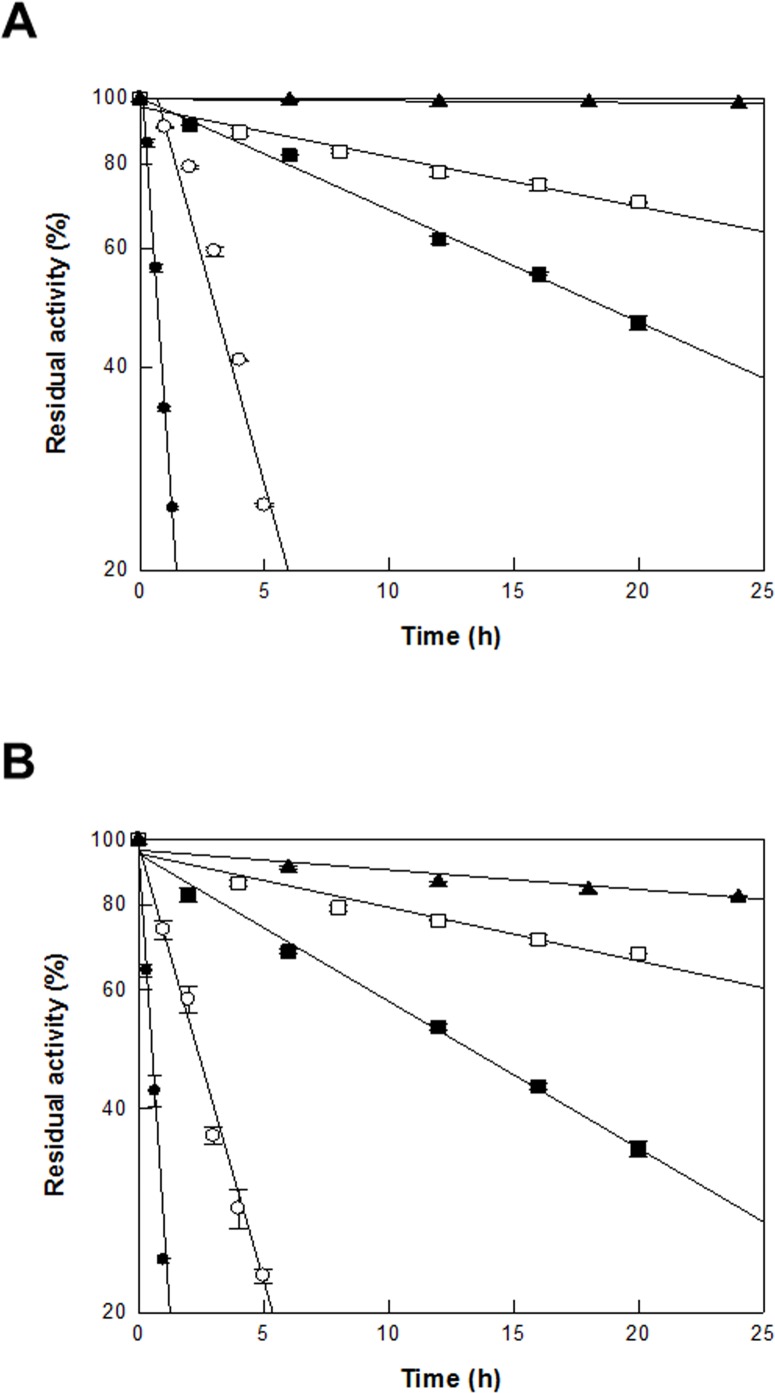
Thermal stability of the activities of DAEase from *F*. *plautii* and DAEase from *F*. *plautii* in recombinant *C*. *glutamicum* cells. **(A) Thermal stability of the activity of recombinant cells.** The permeabilized cells were incubated at 45°C (filled triangle), 50°C (empty square), 55°C (filled square), 60°C (empty circle), and 65°C (filled circle) for various incubation times. **(B) Thermal stability of enzyme activity.** The enzymes were incubated at 45°C (filled triangle), 50°C (empty square), 55°C (filled square), 60°C (empty circle), and 65°C (filled circle) for various incubation times. A sample was withdrawn at each time point and the relative activity was measured. Data represent the means of three separate experiments and error bars represent the standard deviation.

The thermal stabilities of DAEase from *F*. *plautii* in recombinant permeabilized *C*. *glutamicum* cells and the purified enzyme were examined by measuring the activities after incubation at temperatures ranging from 45 to 65°C. Thermal inactivation of the enzyme in cells and the purified enzyme followed first-order kinetics. The half-lives of the enzyme in cells at 45, 50, 55, 60, and 65°C were 9000, 2340, 1098, 211, and 48 min, respectively (Fig 7A), which were 1.8-, 1.0-, 2.3-, 1.4-, and 1.1-fold higher than those of the double-site variant DAEase from *A*. *tumefaciens* [20] in recombinant *E*. *coli* cells, respectively. The half-lives of the purified enzyme at 45, 50, 55, 60, and 65°C were 5760, 2010, 762, 130, and 40 min (Fig 7B), respectively, which were 1.5-, 1.1-, 1.4-, 1.6-, and 1.2-fold lower than those of the enzyme in recombinant *C*. *glutamicum* cells, respectively. Thus, DAEase in cells is more thermally stable than the purified DAEase.

### Effects of cell and substrate concentrations on d-allulose production by permeabilized recombinant *C*. *glutamicum* cells

**Fig 8:**
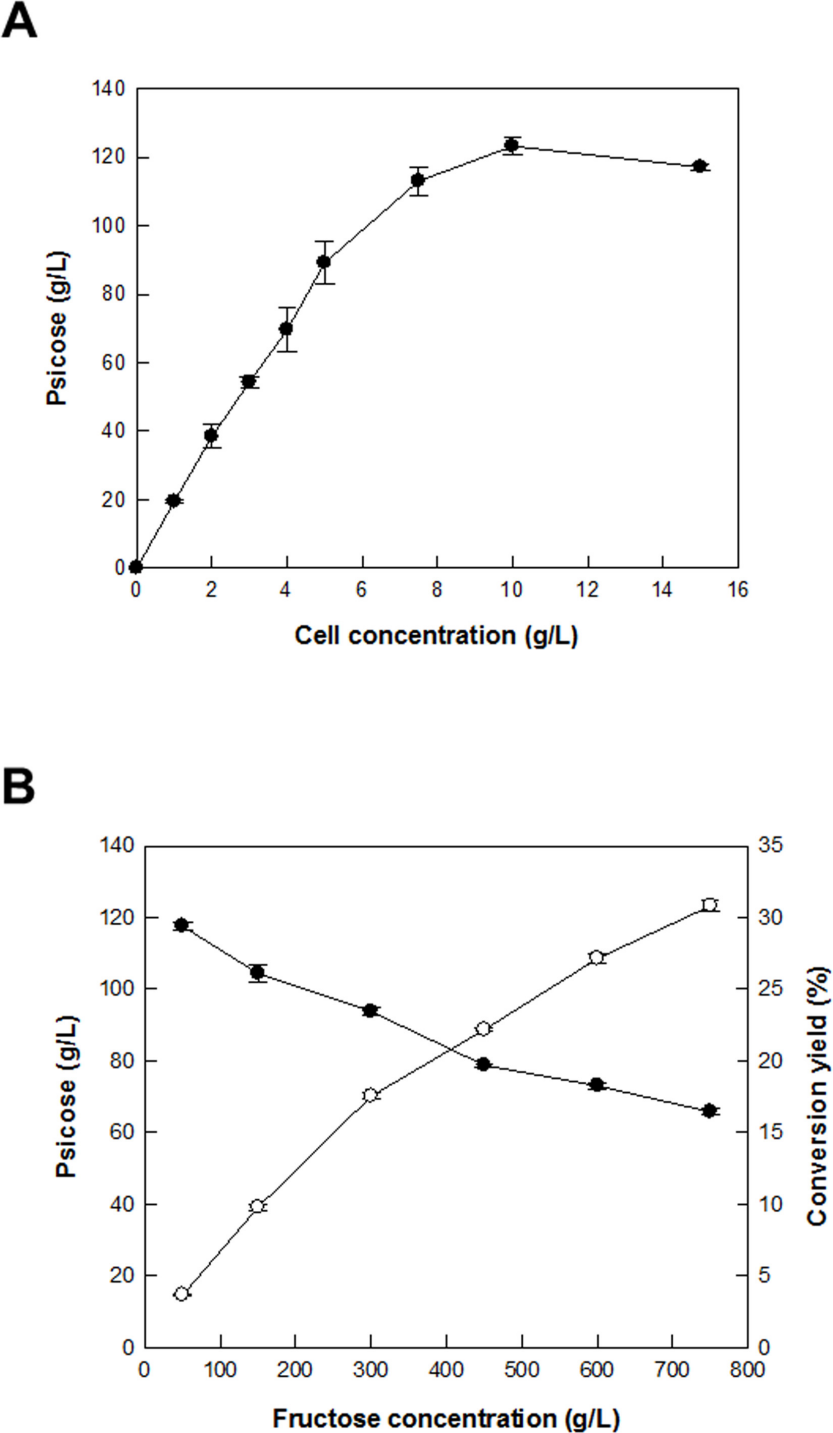
Effects of cell and substrate concentrations on d-allulose production from d-fructose by permeabilized *C*. *glutamicum* cells expressing DAEase from *F*. *plautii*. **(A) Effect of cell concentration.** The reactions were performed by varying the cell concentration from 1 to 15 g/L in 50 mM PIPES buffer (pH 7.5) containing 750 g/L d-fructose in the presence of 1 mM Co^2+^ at 65°C for 10 min. **(B) Effect of substrate concentration.** The reactions were performed by varying the d-fructose concentration from 50 to 750 g/L in 50 mM PIPES buffer (pH 7.5) containing 10 g/L permeabilized cells in the presence of 1 mM Co^2+^ at 65°C for 10 min. Data represent the means of three separate experiments and error bars represent the standard deviation.

The optimal concentration of recombinant permeabilized cells for d-allulose production was determined by varying the concentration of permeabilized cells from 0.5 to 15 g/L with 750 g/L d-fructose as a substrate for 10 min (Fig 8A). Below 10 g/L cells, d-allulose production increased with increasing cell concentration up to 10 g/L cells. However, above 10 g/L cells, the production reached a plateau. Therefore, the cell concentration for the maximal production of d-allulose was 10 g/L. The production of d-allulose from d-fructose was investigated by varying the substrate concentration from 50 to 750 g/L with 10 g/L cells (Fig 8B). As the d-fructose concentration increased, d-allulose production increased, but the conversion rate decreased. To obtain the highest concentration of d-allulose, the concentration of d-fructose as a substrate was determined to be 750 g/L.

### Production of d-allulose from d-fructose by nonpermeabilized and permeabilized recombinant *C*. *glutamicum* cells under the optimized conditions

**Fig 9:**
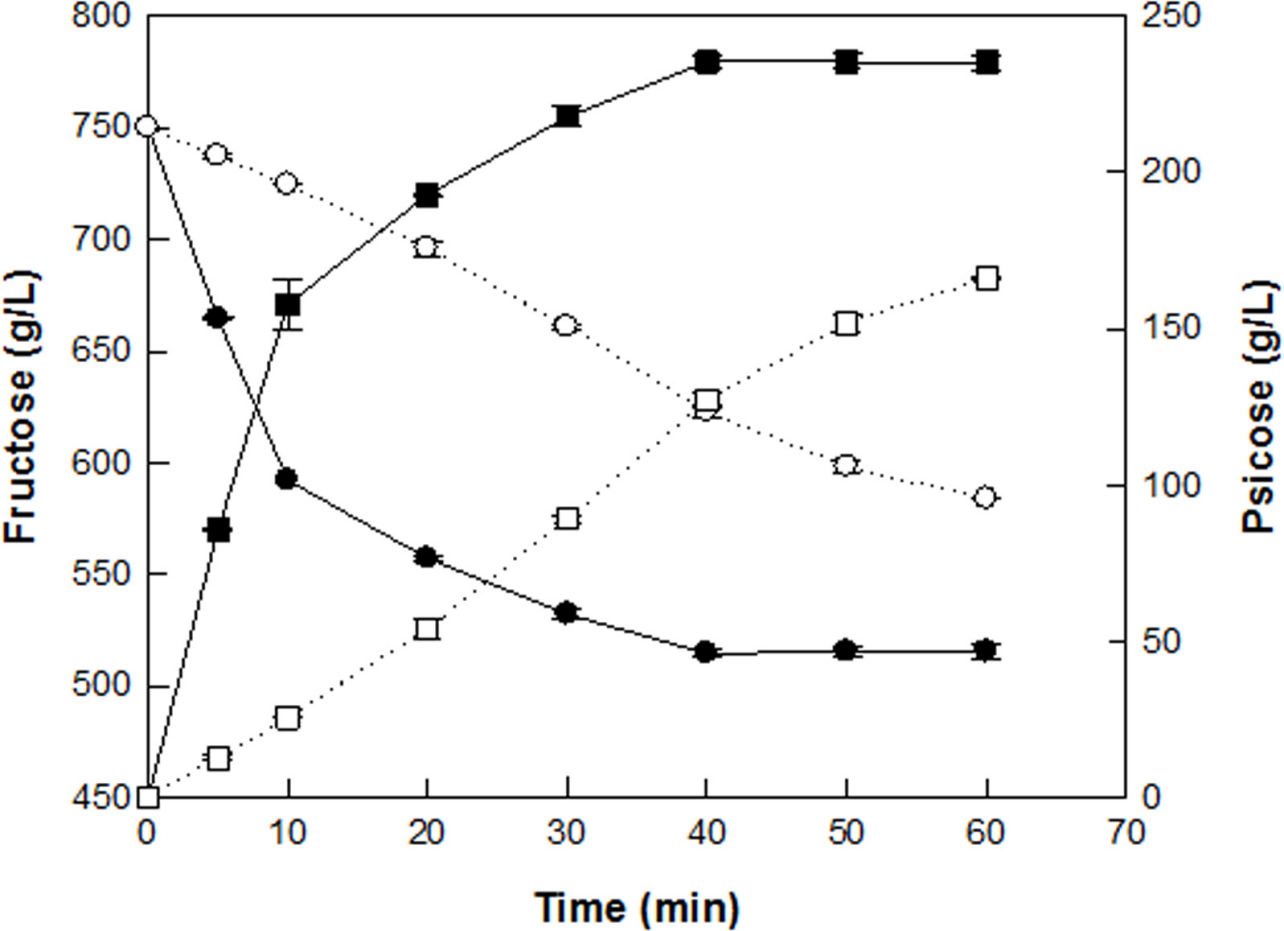
Time-course reactions for the production of d-allulose from d-fructose by permeabilized and nonpermeabilized cells of *C*. *glutamicum* expressing DAEase from *F*. *plautii*. The production of d-allulose (filled square) from d-fructose (filled circle) by permeabilized cells and the production of d-allulose (empty square) from d-fructose (empty circle) by nonpermeabilized cells. The reactions were performed in 50 mM PIPES buffer (pH 7.5) containing 10 g/L cells and 750 g/L d-fructose in the presence of 1 mM Co^2+^ at 65°C for 1 h. Data represent the means of three separate experiments and error bars represent the standard deviation.

The optimal reaction conditions for the production of d-allulose from d-fructose by permeabilized recombinant *C*. *glutamicum* cells expressing DAEase from *F*. *plautii* were pH 7.5, 65°C, 1 mM Co^2+^, 10 g/L cells, and 750 g/L d-fructose. Under the optimized conditions, the time-course reactions for d-allulose production were performed using nonpermeabilized and permeabilized cells (Fig 9). The initial production rate within 10 min of d-allulose by permeabilized cells was significantly higher than that by nonpermeabilized cells. Nonpermeabilized cells produced 166 g/L d-allulose after 60 min, with a conversion rate of 22% (w/w), a specific productivity of 16.6 g/g/h, and a volumetric productivity of 166 g/L/h, whereas permeabilized cells produced 235 g/L d-allulose after 40 min, with a conversion rate of 31% (w/w), a specific productivity of 35.3 g/g/h, and a volumetric productivity of 353 g/L/h, which were 1.4-, 2.1- and 2.1-fold higher than those of nonpermeabilized cells, respectively, and the volumetric productivity of permeabilized cells was 1.5-fold higher than that of purified enzyme. Thus, cell permeabilization was an effective method for increasing d-allulose production.

The permeabilized cells of wild-type *R*. *sphaeroides* treated with 0.1% (w/v) CTAB produced 6.5 g/L d-allulose from 50 g/L d-fructose after 8 h, a specific productivity of 0.03 g/g/h, and a volumetric productivity of 0.81 g/L/h [18]. The permeabilized cells of wild-type *Sinorhizobium* sp. treated with 10% (v/v) toluene produced 37 g/L d-allulose from 700 g/L d-fructose after 15 h, a specific productivity of 0.04 g/g/h, and a volumetric productivity of 2.5 g/L/h [19]. Wild-type cells were not suitable for d-allulose production because of the markedly lower specific productivity than that of recombinant cells. The production of d-allulose from d-fructose by whole recombinant *E*. *coli*, *Bacillus subtilis*, or *C*. *glutamicum* cells expressing DAEase is presented in Table 3. Whole recombinant *E*. *coli* cells expressing the double-site variant (I33L-S213C) DAEase from *A*. *tumefaciens* produced 230 g/L d-allulose from 700 g/L d-fructose after 40 min, with a conversion rate of 33% (w/w), a specific productivity of 86.2 g/g/h, and a volumetric productivity of 345 g/L/h [20], which were previously the highest reported production values. The concentration, volumetric productivity, and thermal stability of recombinant permeabilized *C*. *glutamicum* cells expressing DAEase from *F*. *plautii* in d-allulose production were higher than those of recombinant *E*. *coli* cells expressing the double-site variant DAEase from *A*. *tumefaciens*, whereas the conversion rate and specific productivity were lower. DAEase from *Clostridium scindens* was expressed in *B*. *subtilis* as a food-grade host and the food-grade recombinant *B*. *subtilis* was used for the production of d-allulose [36]. The product concentration, conversion rate, and volumetric productivity of the recombinant *C*. *glutamicum* cells in the resent study were higher than those of the recombinant *B*. *subtilis* cells, but the specific productivity was lower.

**Table 3:** d-Allulose production from d-fructose by whole recombinant cells expressing DAEase.

Host	Strain for enzyme source	Substrate (g l^−1^)	Product (g l^−1^)	Conversion rate (%, w/w)	Half-life at 60°C	Specific productivity (g/g/ h)	Volumetric productivity (g/L/h)	Reference
*E*. *coli*	*Clostridium bolteae*	750	216	29 (55°C, pH 6.5)	NR	40.5	81	[11]
*E*. *coli*	*Clostridium cellulolyticum*	750	218	29 (55°C, pH 8.0)	NR	54.5	109	[12]
*E*. *coli*	*Agrobacterium tumefaciens*	700	230	33 (60°C, pH 8.5)	150	86.2	345	[20]
*B*. *subtilis*	*Clostridium scindens*	700	196	28 (60°C, pH 7.5)	NR	56.0	196	[36]
*C*. *glutamicum*	*Flavonifractor plautii*	750	235	31 (65°C, pH 7.5)	211	35.3	353	This study

NR, not reported.

In conclusion, the putative DAEase from *F*. *plautii* was cloned and expressed in *E*. *coli*. The biochemical properties of the expressed enzyme for the epimerization of d-fructose to d-allulose were characterized. Recombinant *C*. *glutamicum* cells were permeabilized to increase the production of d-allulose. The reaction conditions, including pH, temperature, metal ions, and the concentrations of cells and substrate, were optimized for the permeabilized cells. Under the optimized conditions, the production of d-allulose from d-fructose by permeabilized *C*. *glutamicum* cells expressing DAEase from *F*. *plautii* treated with penicillin and toluene was significantly higher than that by nonpermeabilized cells. Whole permeabilized recombinant *C*. *glutamicum* cells produced d-allulose with the highest concentration and volumetric productivity reported to date.

## Supporting Information

S1 Fig Chromatogram of DAEase from *F*. *plautii* using gel filtration chromatography. The X-axis and Y-axis represent retention time and UV absorbance at 280 nm, respectively. Violet peak represents DAEase, which was eluted at 123 min.
(TIF)

S2 Fig Alignment of the amino acid sequences of DTEase family enzymes. The GenBank accession numbers for DAEases and DTEases are as follows: *F*. *plautii* DAEase (Flpl-DAEase, EHM40452.1), *Dorea* sp. DAEase (Dosp-DAEase, WP_022318236.1), *T*. *primitia* DAEase (Trpr-DAEase, ZP_09717154.1), *Desmospora* sp. DAEase (Desp-DAEase, WP_009711885), *C*. *cellulolyticum* DAEase (Clce-DAEase, ACL75304), *Clostridium* sp. DAEase (Clsp-DAEase, WP_014314767.1), *C*. *scindens* DAEase (Clsc-DAEase, EDS06411.1), *C*. *boltease* DAEase (Clbo-DAEase, EDP19602), *A*. *tumefaciens* DAEase (Agtu-DAEase, AAK88700.1), *Ruminococcus* sp. DAEase (Rusp-DAEase, ZP_04858451.1), *R*. *sphaeroides* DTEase (Rhsp-DTEase, ACO59490), and *P*. *cichorii* DTEase (Psci-DTEase, BAA24429). The metal binding and catalytic residues are appear in blue boxes, and the substrate binding residues appear in red boxes.
(TIF)

S3 Fig Effects of temperature, pH, and metal ions on the activity of DAEase from *F*. *plautii*. **(A) Effect of temperature.** The reactions were conducted by varying the temperature from 30°C to 70°C for 10 min in 50 mM PIPES (pH 7.0) buffer containing 0.5 U/mL enzyme and 50 mM d-fructose in the presence of 1 mM Co^2+^. **(B) Effect of pH.** The reactions were conducted by varying the pH from 6.0 to 8.5 at 65°C for 10 min in 50 mM MES buffer (filled circle, pH 6.0−6.5), 50 mM PIPES buffer (empty circle, pH 6.5−7.5), and EPPS buffer (filled square, pH 7.5−8.5) containing 0.5 U/mL enzyme and 50 mM d-fructose in the presence of 1 mM Co^2+^. **(C) Effect of metal ions.** The reactions were conducted at 65°C for 10 min in 50 mM PIPES (pH 7.0) buffer containing 0.5 U/mL enzyme and 50 mM d-fructose in the presence of 1 mM metal ions. Data are presented as the means of three separate experiments and error bars represent the standard deviation.
(TIF)

S4 Fig Bioconversion of d-allulose from d-fructose by *F*. *plautii* d-allulose 3-epimerase. The reactions were performed in 50 mM PIPES buffer (pH 7.0) containing 8 U/mL enzyme and 750 g/L d-fructose at 65°C for 3 h. Data are presented as the means of three separate experiments and error bars represent the standard deviation.
(TIF)

S5 Fig Effect of combined treatment of permeabilizers on the permeabilization of *Corynebacterium glutamicum* expressing DAEase from *F*. *plautii* for the production of d-allulose from d-fructose. The concentrations of penicillin, span 20, and toluene were 2 mg/L, 1% (w/v), and 5% (v/v), respectively. The reactions were performed in 50 mM PIPES buffer (pH 7.0) containing 7.5 g/L permeabilized cells and 50 mM d-fructose at 65°C for 10 min. Data are presented as the means of three separate experiments and error bars represent the standard deviation.
(TIF)

S6 Fig Effects of temperature and pH on the production of d-psiocse from d-fructose by *Corynebacterium glutamicum* expressing d-psiocse 3-epimerase from *F*. *plautii*. **(A) Effect of temperature.** The reactions were conducted by varying the temperature from 50°C to 75°C for 10 min in 50 mM PIPES (pH 7.5) buffer containing 50 mM d-fructose and 7.5 g/L permeabilized cells. **(B) Effect of pH.** The reactions were conducted by varying the pH from 6.0 to 8.5 at 65°C for 10 min in 50 mM MES buffer (filled circle, pH 6.0−6.5), 50 mM PIPES buffer (empty circle, pH 6.5−7.5), and EPPS buffer (filled square, pH 7.5−8.5) containing 7.5 g/L permeabilized cells and 50 mM d-fructose. Data are presented as the means of three separate experiments and error bars represent the standard deviation.
(TIF)
