# Supplementary figures and images for: D-Allulose Production from D-Fructose by Permeabilized Recombinant Cells of Corynebacterium glutamicum Cells Expressing D-Allulose 3-Epimerase Flavonifractor plautii
Source: PLoS One. 2016 Jul 28;11(7):e0160044. doi: 10.1371/journal.pone.0160044 (PMC4965175; doi:10.1371/journal.pone.0160044)

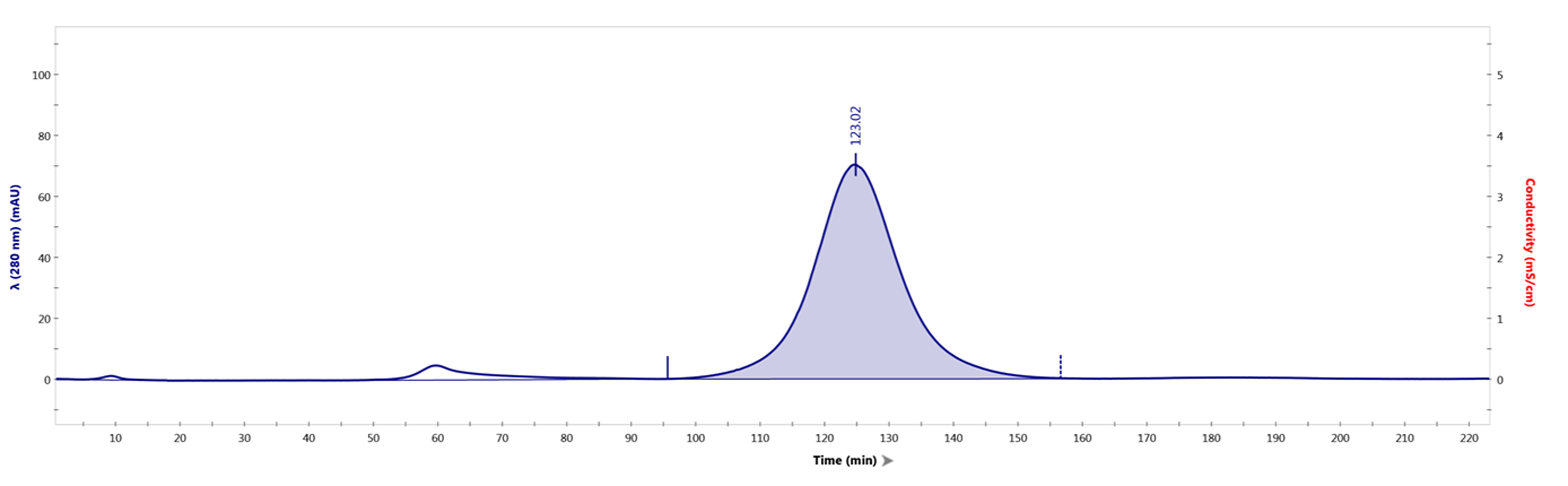

Supplement: S1 Fig — The X-axis and Y-axis represent retention time and UV absorbance at 280 nm, respectively. Violet peak represents DAEase, which was eluted at 123 min. (TIF) [file pone.0160044.s001.tif]

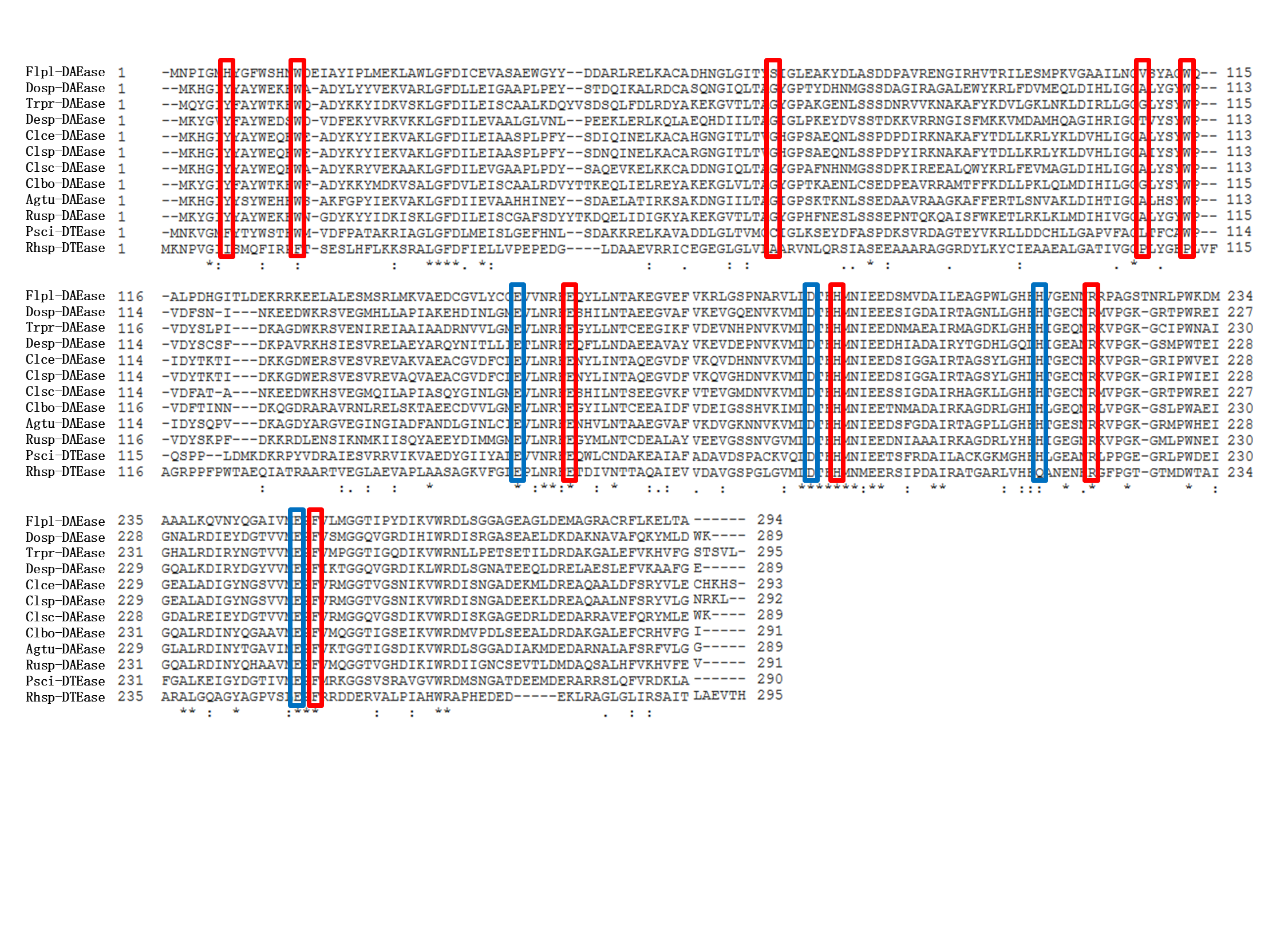

Supplement: S2 Fig — The GenBank accession numbers for DAEases and DTEases are as follows: F. plautii DAEase (Flpl-DAEase, EHM40452.1), Dorea sp. DAEase (Dosp-DAEase, WP_022318236.1), T. primitia DAEase (Trpr-DAEase, ZP_09717154.1), Desmospora sp. DAEase (Desp-DAEase, WP_009711885), C. cellulolyticum DAEase (Clce-DAEase, ACL75304), Clostridium sp. DAEase (Clsp-DAEase, WP_014314767.1), C. scindens DAEase (Clsc-DAEase, EDS06411.1), C. boltease DAEase (Clbo-DAEase, EDP19602), A. tumefaciens DAEase (Agtu-DAEase, AAK88700.1), Ruminococcus sp. DAEase (Rusp-DAEase, ZP_04858451.1), R. sphaeroides DTEase (Rhsp-DTEase, ACO59490), and P. cichorii DTEase (Psci-DTEase, BAA24429). The metal binding and catalytic residues are appear in blue boxes, and the substrate binding residues appear in red boxes. (TIF) [file pone.0160044.s002.tif]

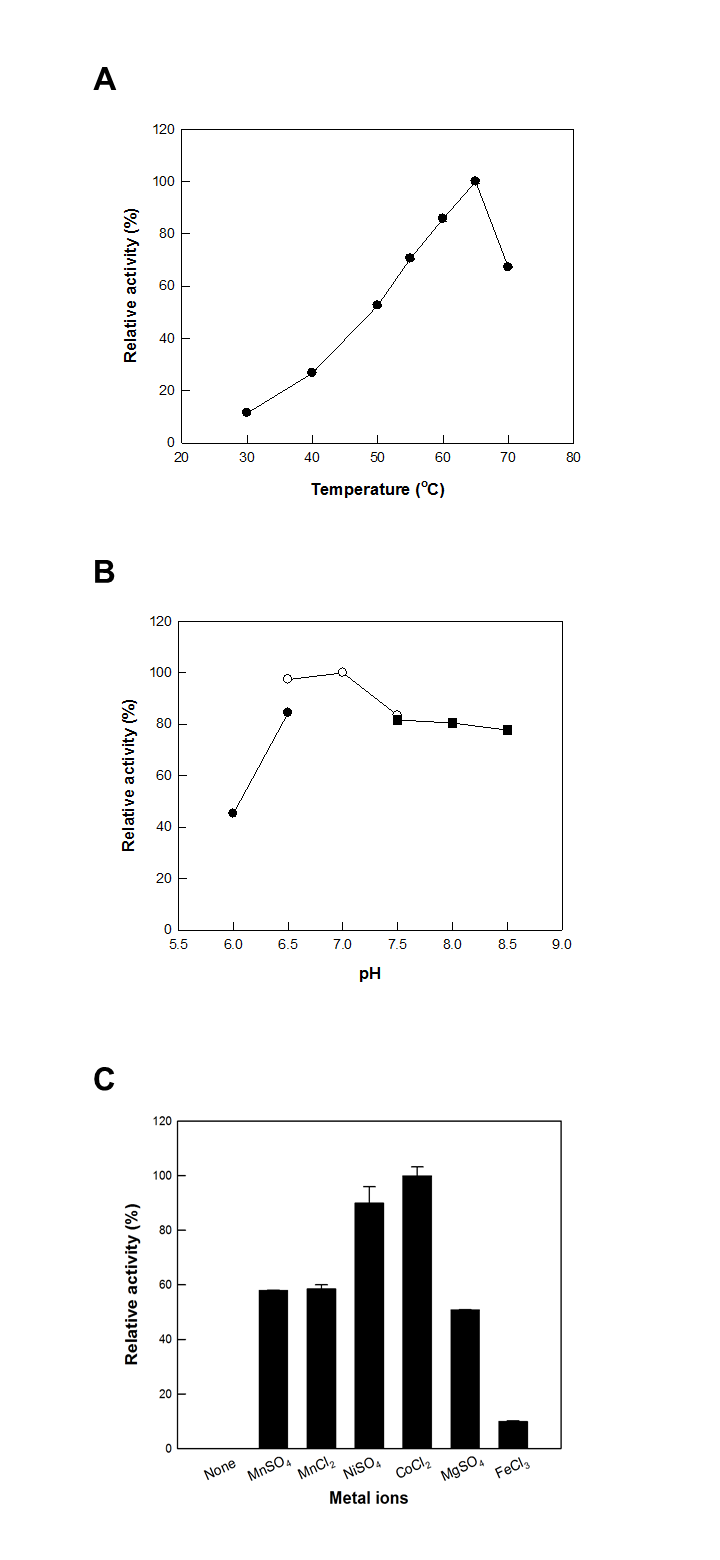

Supplement: S3 Fig — (A) Effect of temperature. The reactions were conducted by varying the temperature from 30°C to 70°C for 10 min in 50 mM PIPES (pH 7.0) buffer containing 0.5 U/mL enzyme and 50 mM d-fructose in the presence of 1 mM Co2+. (B) Effect of pH. The reactions were conducted by varying the pH from 6.0 to 8.5 at 65°C for 10 min in 50 mM MES buffer (filled circle, pH 6.0−6.5), 50 mM PIPES buffer (empty circle, pH 6.5−7.5), and EPPS buffer (filled square, pH 7.5−8.5) containing 0.5 U/mL enzyme and 50 mM d-fructose in the presence of 1 mM Co2+. (C) Effect of metal ions. The reactions were conducted at 65°C for 10 min in 50 mM PIPES (pH 7.0) buffer containing 0.5 U/mL enzyme and 50 mM d-fructose in the presence of 1 mM metal ions. Data are presented as the means of three separate experiments and error bars represent the standard deviation. (TIF) [file pone.0160044.s003.tif]

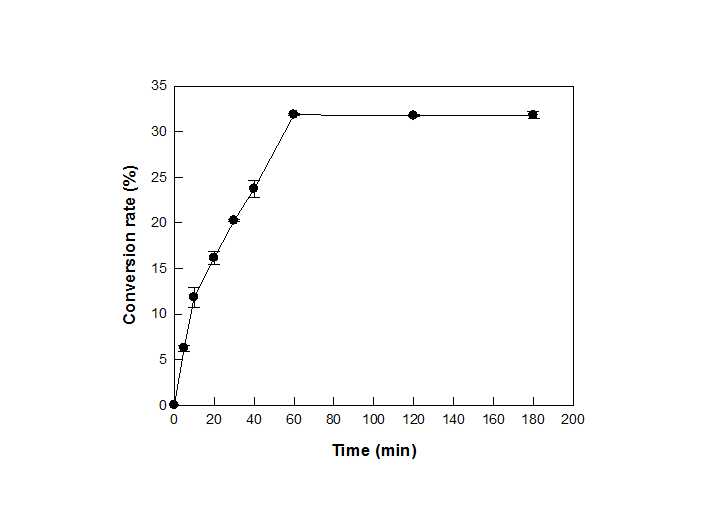

Supplement: S4 Fig — The reactions were performed in 50 mM PIPES buffer (pH 7.0) containing 8 U/mL enzyme and 750 g/L d-fructose at 65°C for 3 h. Data are presented as the means of three separate experiments and error bars represent the standard deviation. (TIF) [file pone.0160044.s004.tif]

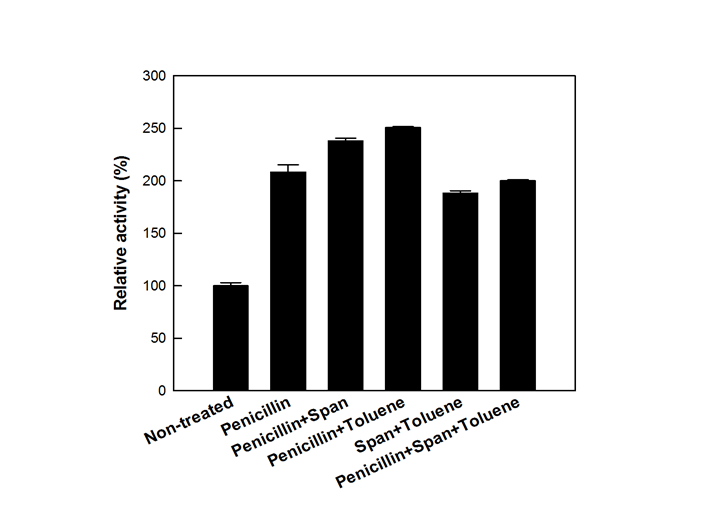

Supplement: S5 Fig — The concentrations of penicillin, span 20, and toluene were 2 mg/L, 1% (w/v), and 5% (v/v), respectively. The reactions were performed in 50 mM PIPES buffer (pH 7.0) containing 7.5 g/L permeabilized cells and 50 mM d-fructose at 65°C for 10 min. Data are presented as the means of three separate experiments and error bars represent the standard deviation. (TIF) [file pone.0160044.s005.tif]

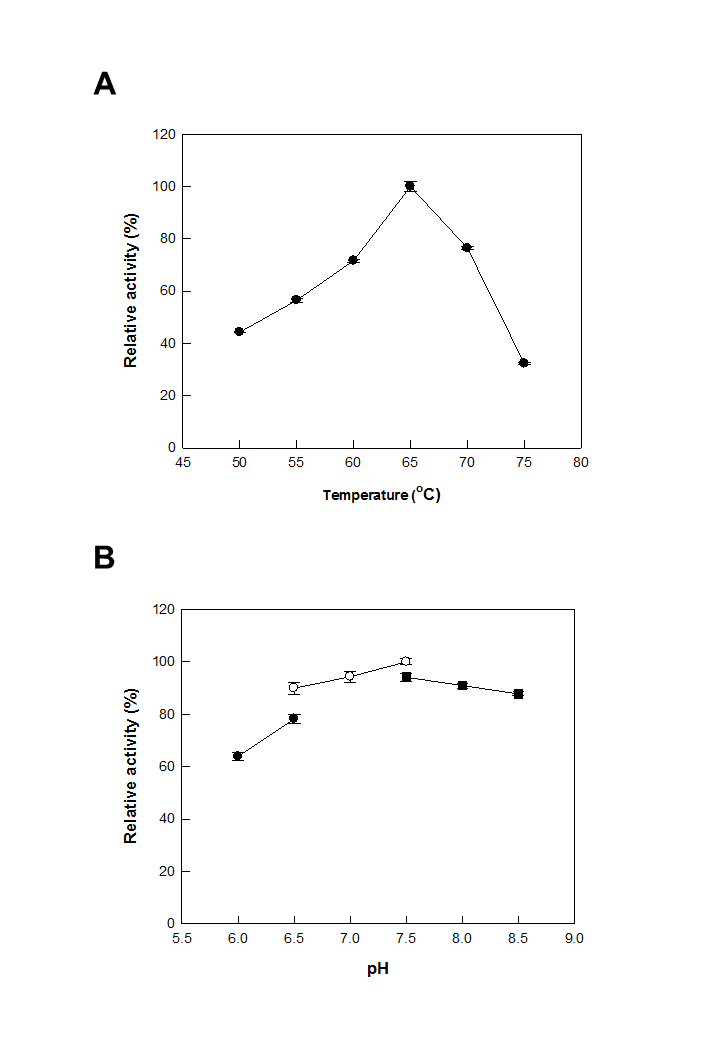

Supplement: S6 Fig — (A) Effect of temperature. The reactions were conducted by varying the temperature from 50°C to 75°C for 10 min in 50 mM PIPES (pH 7.5) buffer containing 50 mM d-fructose and 7.5 g/L permeabilized cells. (B) Effect of pH. The reactions were conducted by varying the pH from 6.0 to 8.5 at 65°C for 10 min in 50 mM MES buffer (filled circle, pH 6.0−6.5), 50 mM PIPES buffer (empty circle, pH 6.5−7.5), and EPPS buffer (filled square, pH 7.5−8.5) containing 7.5 g/L permeabilized cells and 50 mM d-fructose. Data are presented as the means of three separate experiments and error bars represent the standard deviation. (TIF) [file pone.0160044.s006.tif]
